# Migration of cervical spine screws to the sacral canal: a case report

**DOI:** 10.1186/s12883-024-03878-8

**Published:** 2024-10-09

**Authors:** Miao Fang, Yong Zeng, Yueming Song

**Affiliations:** 1 Department of Orthopedics Surgery and Orthopedics Research Institute, West China Hospital of Sichuan University, Chengdu, Sichuan Province 610041 China; 2 Department of Orthopedics Surgery, Chengdu Second People’s Hospital, Chengdu, Sichuan Province 610017 China

**Keywords:** Cervical, Laminoplasty, Implant, Migration, Complication

## Abstract

Cervical open door laminoplasty is widely used in multilevel decompression, which is a motion-sparing decompression treatment option for multilevel cervical myelopathy. Implant distance migration in cervical laminoplasty has not been reported. A 61-year-old woman underwent cervical laminoplasty, three months postoperatively, she experienced left shoulder pain and left upper limb pain, and underwent cervical magnetic resonance imaging, which showed no abnormalities. She gradually developed dizziness, headache, unstable walking, incomplete urinary incontinence, and fluctuating neck lumps. The X-ray showed that the screws of the C7 lateral mass had disappeared and migrated to the sacral canal. The patient underwent cerebrospinal leakage repair and removal of the screws in the spinal canal. Displacement of fixators implanted into the spinal canal after cervical laminoplasty is a rare complication that can cause permanent neurological injury.

## Introduction

Cervical laminoplasty is the most common surgical method performed for treating multilevel cervical spondylosis. The ideal goal is reconstructing the laminar arch to provide sufficient space for the decompressed spinal cord. Traditional forms of laminoplasty fixation, such as suture, may be associated with laminoplasty closure [1]. In cases of suture fixation, up to 34% of patients experienced premature laminoplasty closure [2]. In contrast, the mini plate provides more rigid fixation, allowing patients to move early [6]. A potential drawback of plate laminoplasty is the possibility of screw detachment. However, there have been no reports about complete screw detachment in laminoplasty with plate fixation.

### Case report

**Fig. 1:**
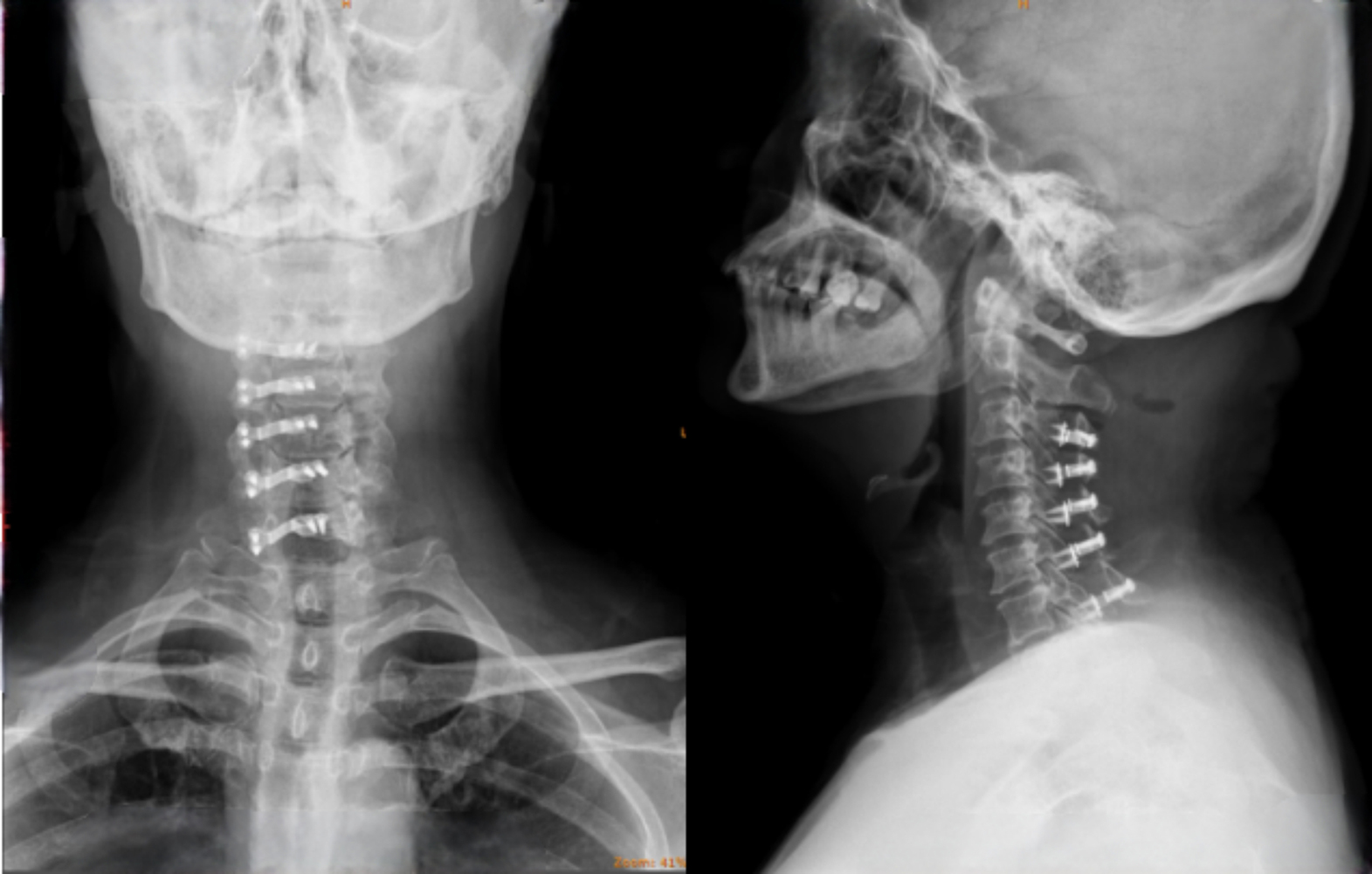
Postoperative anteroposterior X-ray and lateral X-ray showed good positioning of the screws and plates

A 61-year-old woman suffered from pain and numbness in the left shoulder and upper limb due to cervical spondylosis. She underwent cervical laminoplasty with mini plate on June 4, 2019. The operation went well, and the patient’s neurological symptoms improved significantly after the surgery. X-ray confirmed that the position of the internal fixator was good after the surgery (Fig. 1).

However, the patient unexpectedly fell 3 months after surgery and experienced subsequent pain in the left shoulder and upper limb, as well as in the left chest. Nerve injury was suspected, and a cervical magnetic resonance imaging (MRI) examination was conducted, but the results showed no abnormalities. After symptomatic treatment, the pain in the left shoulder, left upper limb, and chest disappeared. The patient subsequently developed dizziness and headache, which were evident during standing and walking, accompanied by numbness in both lower limbs, unstable walking, increased urination frequency and decreased control ability.

**Fig. 2:**
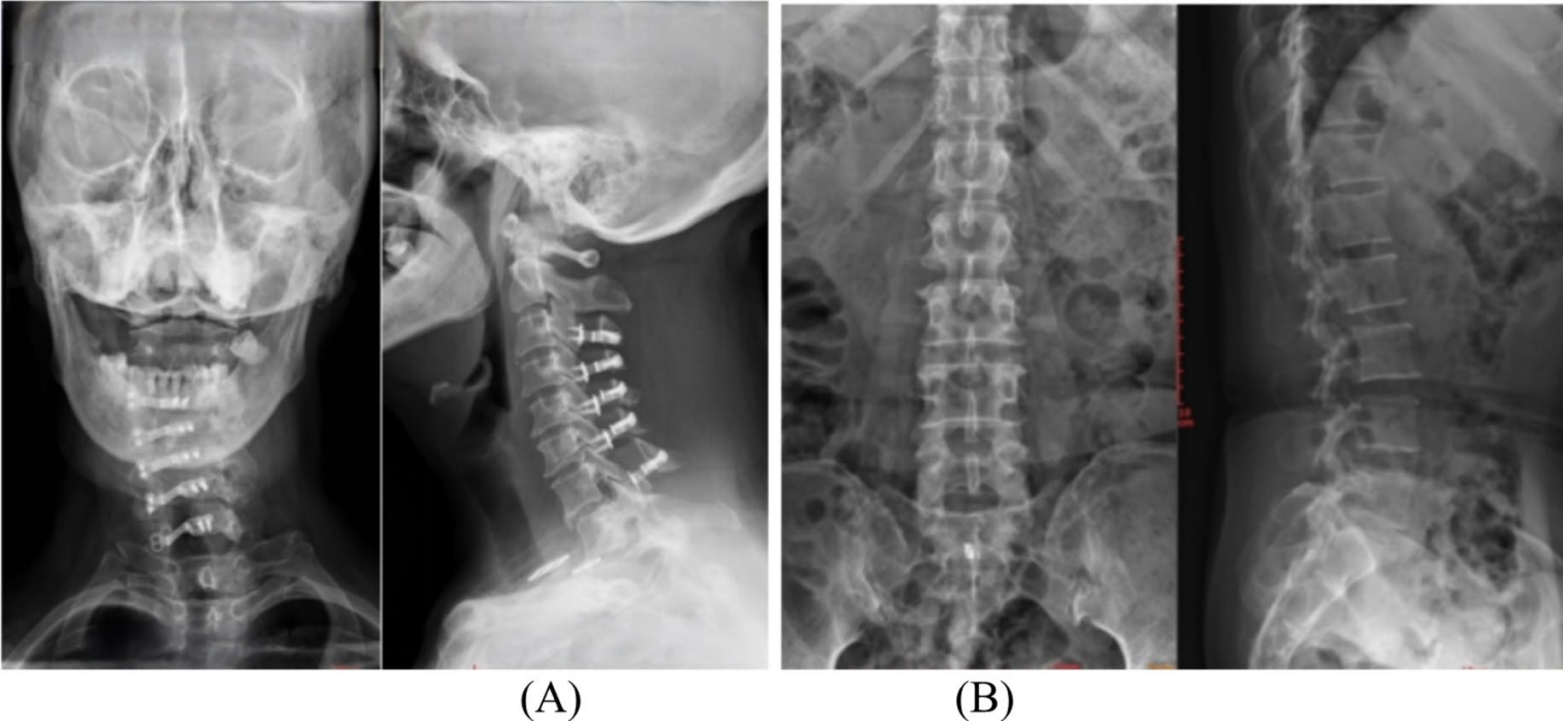
Anteroposterior X-ray and lateral X-rays (**A**) at the 5-month operation showed that the C7 lateral mass screws were missing and that the screws had migrated to the sacral canal (**B**)

Five months after surgery, the patient noticed a fluctuating mass in her neck. Physical examination showed a fluctuating mass in the surgical site. Pressing on a lump caused headaches, the muscle strength in both lower limbs was normal (grade 5/5), and sphincter muscle strength was decreased. Cervical X-ray revealed that the lateral mass screws of C7 had disappeared, and lumbar X-ray showed that the screws had moved to the sacral canal (Fig. 2). Cervical MRI indicated the formation of cerebrospinal fluid cysts in the posterior cervical region.

**Fig. 3:**
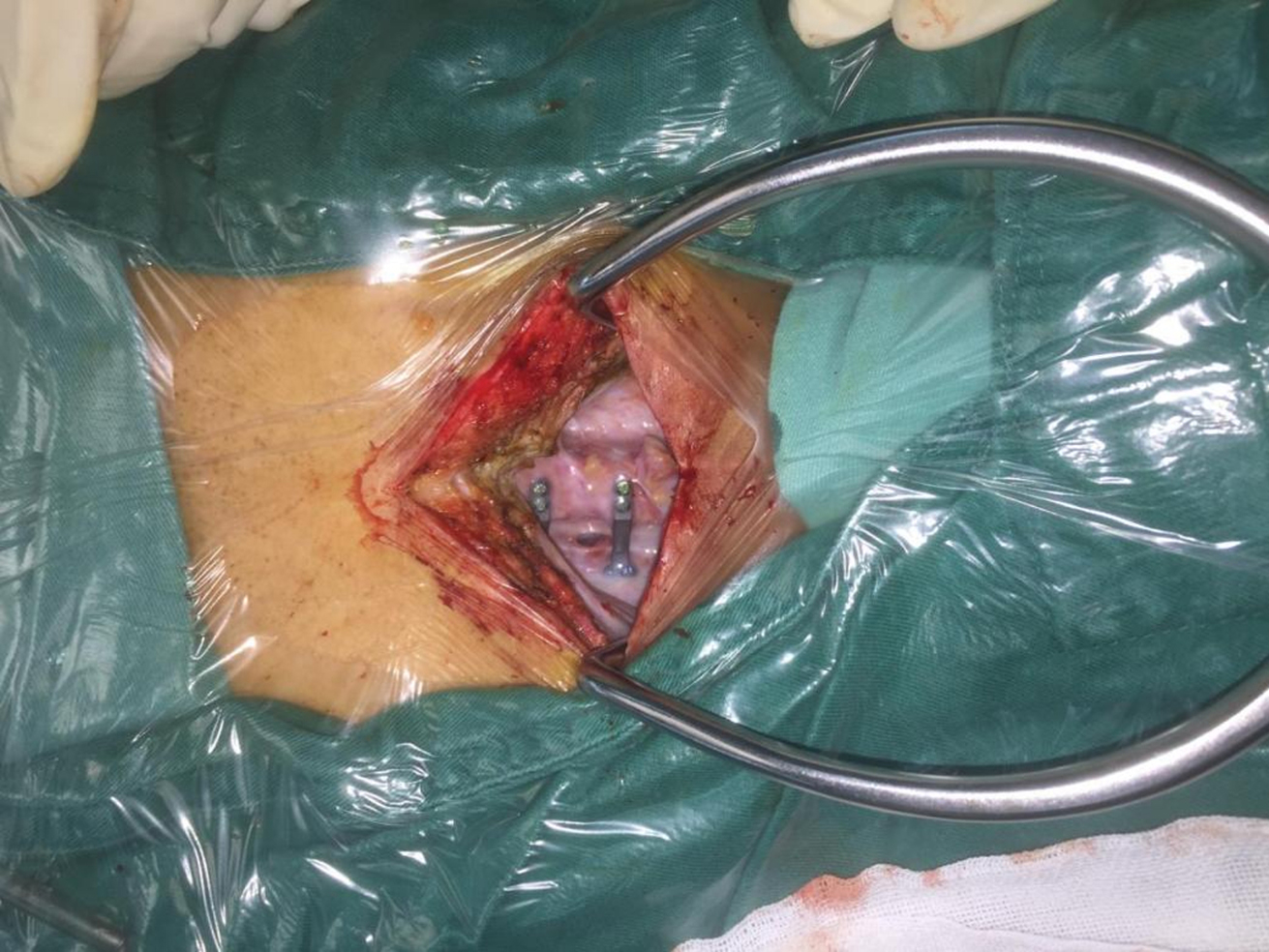
Surgical operation revealed an oval defect in the dura mater on the open side between C6-C7

The patient underwent cerebrospinal fluid leakage repair and the screws that were placed in the sacral canal were removed on November 6, 2019. During the operation, a cerebrospinal fluid cyst was found in the posterior neck. An oval defect was in the dura mater on the open side between C6-C7, and the screws of the C7 lateral mass disappeared (Fig. 3). The C7 laminar was not closed, and the mini plate was not loosened. After the surgery, her symptoms of dizziness, headache, and unstable walking disappeared, but there was some decrease in sphincter ability. After a follow-up of 46 months, the surgical site was normal, lower limb numbness still existed, and sphincter function was still not fully normal.

## Discussion

Cervical open door laminoplasty is widely used in multisegment decompression, which is a motion-sparing decompression treatment option for multilevel cervical myelopathy [3]. Based on Hirabayashi’s original method, the laminae were kept open by stay sutures [4]. Although it is a simple and cost-effective surgical method, sutures do not provide rigid fixation. Loose fixation is a serious problem in closing open laminae in that it may result in suboptimal decompression of the spinal cord [5]. Park and Heller designed a mini plate for cervical laminoplasty, the plate can primarily provide immediate fixation in cervical laminoplasty. The use of this plate will allow the patient to engage in an early active rehabilitation protocol-while minimizing the risk of restenosis of the canal [6]. Although this technology has been clinically applied for several years without any reports of implant displacement, but there is also a potential risk of screw and plate loosening and falling off.

The patient had an accidental fall three months after surgery, and she developed pain in her left shoulder and upper limbs. Cervical MRI did not reveal any abnormalities. Then, she exhibited symptoms of low intracranial pressure and spinal cord stimulation. Five months postoperatively, the patient developed a pulsating mass in the neck, which received further attention. The imaging demonstrated that the screw had moved into the sacral canal.

The mechanism of screw detachment in our case was unclear. We speculated that the shear force transmitted to the screw through the plate caused the screw to loosen when the patient suffered trauma. The repeated rotational movements of the cervical spine caused the screws to completely back out. Due to body position and other reasons, the posterior part of the neck was compressed, coupled with dural pulsation. The dura mater and arachnoid membrane were punctured by sharp screws. Then, the screw entered the subarachnoid space and moved toward the sacral canal due to both body position and gravity. After the rupture of the arachnoid membrane, the continuous pressure of cerebrospinal fluid acts on the neck, resulting in the formation of cerebrospinal fluid cysts.

John M et al. reported that when reconstructing the stability of the cervical lamina using mini plate, approximately 2.3% of the segments experienced screw back-out, but there was no reports of looseness in the medical records of patients who underwent the procedure with two screws [1]. Patients who experience displacement of spinal implants may experience no symptoms or require emergency intervention depending on the geometric shape, size, path of migration, tissue tension, and degree of tissue deformability of the displaced object. In our case, the subarachnoid buffer space was limited, and the spinal cord and cauda equina nerve had poor resistance to injury. Therefore, the screw entered the subarachnoid space and caused damage to the spinal cord and cauda equina nerve during the descent process. Although the displaced implant was surgically removed, there were still residual neurological sequelae after the surgery.

## Conclusion

The possibility of screw displacement after laminoplasty cannot be ignored. Although the situation is rare, implants in laminoplasty can migrate from the spine to a distant location, which may result in serious consequences.

## Data Availability

No datasets were generated or analysed during the current study.
